# Bacterial contamination level and characterization of antimicrobial-resistant bacteria in commercial pet foods in Japan

**DOI:** 10.1016/j.onehlt.2025.101197

**Published:** 2025-09-10

**Authors:** Akira Fukuda, Kazu Yamaura, Kazuyoshi Tokumoto, Yasuhiko Suzuki, Chie Nakajima, Shoichiro Yukawa, Masaru Usui

**Affiliations:** a Food Microbiology and Food Safety Unit, Division of Preventive Veterinary Medicine, School of Veterinary Medicine, Rakuno Gakuen University, Ebetsu, Japan; b Harmony Corporation, Tokyo, Japan; c Division of Bioresources, Hokkaido University International Institute for Zoonosis Control, Sapporo, Hokkaido, Japan; d International Collaboration Unit, International Institute for Zoonosis Control, Hokkaido University, Hokkaido, Japan; e Division of Research Support, Hokkaido University Institute for Vaccine Research and Development, Sapporo, Hokkaido, Japan; f Vaccinology for Clinical Development, Hokkaido University Institute for Vaccine Research and Development, Sapporo, Hokkaido, Japan; g Department of Comparative Animal Science, College of Life Science, Kurashiki University of Science and The Arts, Okayama, Japan

**Keywords:** Antimicrobial-resistant bacteria, Bacterial contamination, Cephalosporin, *Escherichia coli*, *Enterococcus*, Linezolid, Pet food

## Abstract

With the growing pet market, various types of pet food become available. Pet foods, especially raw meat-based diets (RMBDs), are contaminated with pathogens and antimicrobial-resistant bacteria, leading to health concerns for pets and humans. The bacterial contamination levels in pet food, including the presence of antimicrobial-resistant bacteria, have not been extensively studied in Japan. To address this issue in commercial pet foods in Japan, we evaluated the standard plate counts (SPCs) of various pet food samples, and isolated and characterized *Escherichia coli* and *Enterococcus* spp. A total of 129 pet foods (48 RMBDs, 21 heat-treated foods, and 60 treats) were purchased, and SPC quantification and isolation of *E. coli* and *Enterococcus* spp. were performed. SPCs in RMBD were significantly higher than in heat-treated foods and treats. Specifically, 50.0 % of the RMBD samples had SPCs exceeding 10^6^ CFU/g. *E. coli* was isolated only from 62.5 % of RMBDs. Four cephalosporin-resistant *E. coli* strains harboring *bla* genes were detected, among which, one carried *mcr* conferring resistance to colistin. *Enterococcus* spp. were isolated from 89.6 % of RMBDs and 23.3 % of treats. Linezolid-non-susceptible *E. faecalis* harboring *optrA* or *poxtA* genes were detected in four RMBDs. Pet food may be contaminated with bacteria; some RMBDs are contaminated at concentrations >10^6^ CFU/g, and with veterinary and human clinically important antimicrobial-resistant bacteria. To prevent health risks in both humans and pets associated with pet food, ensuring the hygienic management of pet food, especially RMBDs, and promoting accurate knowledge among pet owners are important.

## Highlights

• Half of raw meat-based diets for pets exceed 10^6^ CFU/g in standard plate count.
• *Escherichia coli* and enterococci were isolated from 62.5 % and 89.6 % of raw pet foods.
• Cephalosporin- and colistin-resistant *Escherichia coli* was detected in raw pet foods.
• *optrA*- and *poxtA*-harboring *Enterococcus faecalis* were detected in raw pet foods.

## 1 Introduction

With the increasing number of pets, the pet industry is continuously growing internationally [1]. Various types of pet food are commercially available. Canned and kibble pet foods undergo heating at high temperatures, dehydrated and freeze-dried processing methods are also employed. In contrast, raw pet foods, known as raw meat-based diets (RMBDs), typically comprise uncooked animal meats [2]. Notably, various adverse health issues have been reported in pets and humans due to pathogen contamination of pet foods [3,4]. Therefore, appropriate hygiene management with microbiological control in pet food production are essential to ensure public health.

As the development of mail orders and supply chains expanded, the market size of RMBDs grew and feeding RMBDs to pets increased [5,6]. RMBDs commonly contain ingredients such as chicken and meat from wild animal meat. Due to their lack of heat treatment, RMBDs are frequently contaminated with animal-derived bacteria, raising concern regarding potential bacterial transmission [1,4,7]. To address this issue, the Centers for Disease Control and Prevention (CDC) has recommended against feeding RMBDs to pets [2]. It is therefore necessary to assess the extent of RMBD contamination. However, in Japan, only a few studies have investigated the contamination status of *Salmonella* spp. in pet foods, and bacterial contamination levels in commercial pet foods, including RMBDs, remain unclear [8,9].

The transmission of antimicrobial-resistant bacteria between humans and animals has become a growing concern from a One Health perspective. Antimicrobial-resistant bacteria of clinical importance in human and veterinary medicine, including cephalosporin-resistant *Escherichia coli* and linezolid-resistant *Enterococcus* spp., have been isolated from pet foods [[10], [11], [12]]. Cephalosporins are classified as highest priority critically important antimicrobials used in veterinary and human clinical settings [13]. Linezolid is a last-resort antimicrobial exclusively approved for treating multidrug-resistant gram-positive bacterial infections use in humans [14]. Feeding of pet foods contaminated with antimicrobial-resistant bacteria increased the risks of pets harboring antimicrobial-resistant bacteria [15]. Moreover, transmission of microorganisms from pet foods and pets to humans has been reviewed [16].

To prevent the dissemination of bacteria, including antimicrobial-resistant bacteria, from pet foods, investigating and understanding the actual state of bacterial contamination in these products, especially in RMBDs, is crucial. In this study, we evaluated the standard plate counts (SPCs) of various pet food types and, isolated and characterized *E. coli* and *Enterococcus* spp. as fecal contamination indicator bacteria from these food sources.

## 2 Materials and methods

### 2.1 Sampling

A total of 129 pet foods, comprising 48 RMBDs (30 raw meats and 18 bones and raw foods [BARFs]), 21 heat-treated foods, and 60 treats, purchased by mail order or from supermarkets between March 2022 and March 2023 (Table S1). Pet food products were randomly purchased, and duplication of the same items was checked to ensure none occurred. RMBDs are sold frozen and are prepared using various types of meat as the main ingredients. Raw meats consisted of sliced or minced raw muscle of meat. BARFs were formulated from raw ingredients, including muscle, organ meat, and bone, sourced from domestic and wild animals, and other materials, such as grains and vegetables [17]. Heat-treated foods, namely kibbles (*n* = 13), which were dried, and canned foods (*n* = 8) with high moisture content, were sealed after heat processing. Treats were dried and/or heated and sold as snacks for pets. After purchase, the products were stored and processed in accordance with the manufacturer's instructions.

### 2.2 Quantification and isolation of bacteria

Each sample (25 g) was added to 225 mL of phosphate-buffered saline and left at room temperature for 30 min. The mixture was homogenized at 200 rpm for 5 min using a stomacher. The homogenate was utilized for SPC quantification and isolating *E. coli* and *Enterococcus* spp.. SPC was conducted according to the inspection methods for pet food [18]. The homogenate was diluted using phosphate-buffered saline and 1 mL of the diluent was added to 20 mL of standard agar medium (Nissui Pharmaceutical, Co., Ltd. Tokyo, Japan), incubated at 36 °C for 48 h, and the number of colonies was counted. To isolate *E. coli*, the homogenate was plated onto CHROMagar™ECC agar (CHROMagar, Paris, France) with/without 1 mg/L cefotaxime (Sigma-Aldrich, MO, USA), and incubated at 36 °C overnight. To isolate *Enterococcus* spp., the homogenate was plated onto Enterococcosel agar (Becton, Dickinson and Company, Franklin Lakes, NJ, USA) with/without 16 mg/L florfenicol (Sigma-Aldrich), and incubated at 36 °C for 24–48 h. Up to two colonies were picked from a single plate. When two isolates from the same plate exhibited the same bacterial species and antimicrobial susceptibility profiles, they were considered as single isolates.

### 2.3 Bacterial identification

The bacterial species of each isolate was identified using matrix-assisted laser desorption/ionization time-of-flight mass spectrometry using a MALDI Biotyper system (Bruker Daltonics, Bremen, Germany) [14]. Additionally, PCR with species-specific primers based on the superoxide dismutase (*sodA*) gene was used to identify species of *Enterococcus* spp. [19].

### 2.4 Antimicrobial susceptibility testing

Antimicrobial susceptibility was determined using the agar dilution method, with the exception of colistin for *E. coli*, which was determined using the microbroth dilution method. The resistance breakpoints were defined in accordance with the Clinical Laboratory Standards Institute guidelines, except for tigecycline for *E. coli*, and florfenicol for *Enterococcus* spp., the breakpoints of which were not defined [20,21]. The clinical breakpoint of tigecycline for *E. coli* in European Committee on Antimicrobial Susceptibility Testing (EUCAST) (https://www.eucast.org/clinical_breakpoints) and the epidemiological cut-off values of florfenicol for *Enterococcus* spp. in minimum inhibitory concentration (MIC) EUCAST (https://mic.eucast.org/) were applied to *E. faecium* and *E. faecalis*, with MIC of florfenicol >8 mg/L indicating resistance. *E. coli* ATCC25922, *Pseudomonas aeruginosa* ATCC27853, *E. faecalis* ATCC29212, and *Staphylococcus aureus* ATCC29213 were used as quality control strains.

### 2.5 Detection of antimicrobial resistance genes

Antimicrobial resistance genes were screened based on antimicrobial susceptibility phenotypes. For cefotaxime-resistant *E. coli* isolates, *bla* genes were analyzed via PCR, and *bla*_CTX-M_ gene subtypes were subsequently identified via sequencing, as previously described [[22], [23], [24]]. *E. coli* isolates exhibiting MICs of colistin ≥2 mg/L were analyzed for the presence of *mcr* genes via PCR [25,26]. For linezolid-non-susceptible (resistant or intermediate) *Enterococcus* spp. isolates, mobile linezolid resistance genes (*optrA*, *poxtA*, and *cfr*) were detected via PCR [27,28].

### 2.6 Whole-genome sequencing and bioinformatics analysis

Whole-genome short-read sequencing was performed for *bla*_CTX-M_- and *mcr-1*-positive *E. coli* and *optrA*- or *poxtA*-positive *Enterococcus* spp. isolates. For short-read sequencing, genomic DNA was extracted from *E. coli* and *Enterococcus* spp. using the QIAquick PCR Purification Kit and DNeasy Blood & Tissue Kit (Qiagen, Hilden, Germany), respectively. The contigs were mapped with 150 bp paired-end reads obtained using NEBNext® Ultra™ II FS DNA (New England Biolabs, MA, USA) and NovaSeq sequencing platforms (Illumina, San Diego, CA, USA). Illumina reads were assembled de novo using Shovill v1.1.0 with default parameters (https://github.com/ablab/spades). Antimicrobial resistance genes were identified using ResFinder v4.1 (https://github.com/cadms/resfinder). Multilocus sequence typing (MLST) was performed using the Center for Genomic Epidemiology server (http://www.genomicepidemiology.org) and pubMLST (https://pubmlst.org) to determine the sequence type (ST).

### 2.7 Data analysis

Statistical significance in SPCs between types of pet foods (raw meats, BARFs, heat-treated foods, and treats) was determined using the Mann-Whitney *U* test, with the significance threshold set at *p* < 0.05.

## 3 Results

### 3.1 SPC

**Fig. 1:**
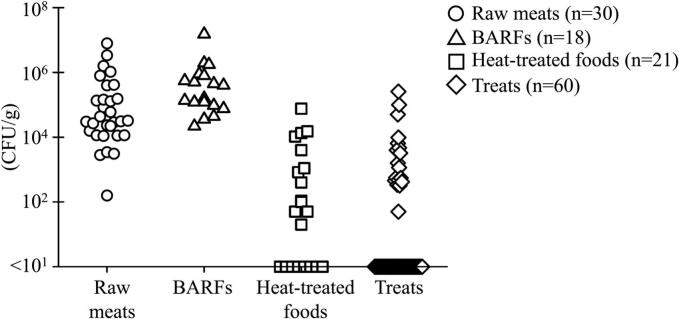
SPCs of pet foods. The detection limit was set to above 10 CFU/g. Statistically significant differences were observed among all sample types.

The average SPCs in raw meats, BARFs, heat-treated foods, and treats were 5.6 × 10^5^, 1.3 × 10^6^, 5.8 × 10^3^, and 7.4 × 10^3^ CFU/g, respectively (Fig. 1). SPCs were significantly higher in the order of BARFs, raw meats, treats, and heat-treated foods. In all raw meats and BARFs, SPCs were countable, exhibiting ranges of 1.6 × 10^2^–8.0 × 10^6^ and 2.2 × 10^4^–1.5 × 10^7^ CFU/g, respectively. Notably, 50 % (24/48) of RMBDs (36.7 % [11/30] raw meats and 72.2 % [13/18] BARFs) exceeded 10^6^ CFU/g. Meanwhile, 61.9 % (13/21) of heat-treated foods and 30.0 % (18/60) of the treats had SPCs above the detection limit (≥ 10 CFU/g) with ranges of 2.0 × 10^1^ to 7.5 × 10^4^ CFU/g and 5.0 × 10^1^ to 2.6 × 10^5^, respectively.

### 3.2 Isolation and characterization of *E. coli*

*E. coli* strains were isolated from 62.5 % (30/48) of the RMBDs (53.3 % [16/30] raw meats and 77.8 % [14/18] BARFs) but not from heat-treated foods and treats (Table 1). Ampicillin-, cefotaxime-, tetracycline-, ciprofloxacin-, kanamycin-, and colistin-resistant *E. coli* strains were detected in nine (30.0 %), four (13.3 %), four (13.3 %), three (10.0 %), three (10.0 %), and one (3.3 %) RMBDs, respectively. All *E. coli* isolates were susceptible to fosfomycin, meropenem, and tigecycline. In four cefotaxime-resistant *E. coli* strains from four RMBDs (two raw meats and two BARFs) containing chicken as the major component, two *bla*_CTX-M-2_-, one *bla*_CTX-M-14_-, and one *bla*_CMY-2_-positive strains were detected. One of these strains (60C1) was resistant to colistin and carried the *mcr-1* gene. Whole-genome analysis was performed for both the cefotaxime- and colistin-resistant *E. coli* strains (60C1) (Table 2). *E. coli* 60C1 showed resistance to ampicillin, cefotaxime, kanamycin, and colistin, and harbored related resistance genes (*bla*_CTX-M-14_, *mcr-1,* and *aph(3′)-IIa*). The ST of *E. coli* 60C1 was identified as ST1084 in the MLST database.

**Table 1:** Isolation of *E. coli* and antimicrobial resistance in RMBDs.

Main meat (protein) source	Number of raw diets	Isolation of *E. coli (%)*	Isolation of antimicrobial-resistant *E. coli* (%)					
			Ampicillin	Cefotaxime	Tetracycline	Ciprofloxacin	Kanamycin	Colistin
Total	48	30	9	4	4	3	3	1
		(62.5 %)	(30.0 %)	(13.3 %)	(13.3 %)	(10.0 %)	(10.0 %)	(3.3 %)
Chicken	11	10	6	4	3	2	3	1
Deer	11	5						
Horse	9	5	1		3	1		
Cow	4	3						
Sheep	3	3	1					
Fish	2	1						
Kangaroo	2	1						
Mixed^a^	1	1	1		1			
Boar	1							
Crocodile	1							
Duck	1							
Ostrich	1	1						
Pig	1							

Resistance to fosfomycin, meropenem, and tigecycline was not detected.

a Mixed contained cow, sheep, chicken, and pig meat.

**Table 2:** Characterization of *bla*_CTX-M-14_- and *mcr-1*-positive *E. coli* strains.

Strain No.		60C1
Sample No.		60
Food Category		BARFs
Major components		chicken (meat, liver, heart), vegetables, fruits, alfalfa
Minimum inhibitory concentration (mg/L)	Ampicillin	**>128**
	Cefotaxime	**>64**
	Tetracycline	1
	Ciprofloxacin	<0.03
	Kanamycin	**>128**
	Colistin	**4**
	Fosfomycin	0.25
	Meropenem	<0.06
	Tigecycline	0.25
Antimicrobial resistance genes		*bla*_CTX-M-14_, *mcr-1, aph(3′)-IIa*
ST		1084
Accession No.		SAMD00915481

Resistance is indicated in underlined bold.

ST: Sequence type.

### 3.3 Isolation and characterization of *Enterococcus* spp.

*Enterococcus* spp. strains were isolated from 89.6 % (43/48) of RMBDs (83.3 % raw meats [25/30] and 100 % [18/18] BARFs), 23.3 % (14/60) of treats, and no heat-treated foods (Table 3). Tetracycline-, erythromycin-, and florfenicol-resistant *Enterococcus* spp. strains were detected in 14 (32.6 %), 10 (23.3 %), and 4 (9.3 %) RMBDs, respectively. Linezolid-intermediate *Enterococcus* spp. strains were detected in four (9.3 %) RMBDs. In one (7.1 %) treat, tetracycline-resistant *Enterococcus* spp. was detected. All *Enterococcus* spp. strains were susceptible to ampicillin, ciprofloxacin, and vancomycin. Among the four linezolid-intermediate *E. faecalis* from RMBDs, *optrA* or *poxtA* genes were detected. Whole genome sequencing revealed that the four strains were resistant to florfenicol and carried either *fexA* or *fexB* genes (Table 4). Three strains were resistant to erythromycin and tetracycline and carried related antimicrobial resistance genes (*ermA* or *ermB* and *tet(L)*). The ST types of these four strains were identified as ST1071, ST32, and ST960 in the MLST database.

**Table 3:** Isolation of *Enterococcus* spp. and antimicrobial resistance in pet foods.

Food types	Main meat (protein) source	Number of raw diets	Isolation of *Enterococcus* spp.	Isolation of antimicrobial-resistant *Enterococcus* spp. (%)			
				Tetracycline	Erythromycin	Florfenicol	Linezolid^b^
RMBDs	Total	48	43	14	10	4	4
			(89.6 %)	(32.6 %)	(23.3 %)	(9.3 %)	(9.3 %)
	Chicken	11	11	7	6	1	1
	Deer	11	8	2	1		
	Horse	9	7			1	1
	Cow	4	4	1	1		
	Sheep	3	3				
	Fish	2	2				
	Kangaroo	2	2				
	Mixed^a^	1	1	1	1	1	1
	Boar	1	1				
	Crocodile	1	1				
	Duck	1	1	1			
	Ostrich	1	1	1			
	Pig	1	1	1	1	1	1
Treats	Total	60	14	1			
			(23.3 %)	(7.1 %)			
	Chicken	41	10	1			
	Cow	5	1				
	Fish	5	1				
	Mixed^a^	1	1				
	Boar	1					
	Pig	1	1				
	Non-meat base	6					

Resistance to ampicillin, ciprofloxacin, and vancomycin was not detected.

RMBD: Raw meat-based diet.

a Mixed contained cow, sheep, chicken, and pig meat.

b Four linezolid-intermediate strains were identified.

**Table 4:** Characterization of *optrA*- or *poxtA*-positive *E. faecalis* strains.

Strain No.		9P1	10P1	60P2	61P2
Sample No.		9	10	60	61
Food category		BARFs	BARFs	BARFs	BARFs
Major components		pig (meat, heart, liver, kidney), vegetables, fruits, alfalfa	cow (meat, heart, kidney), sheep meat, chicken meat, pig meat, vegetables, fruits, alfalfa	chicken (meat, liver, heart), vegetables, fruits, alfalfa	horse (meat, liver, heart, kindly), chicken, vegetables, fruits, alfalfa
Species		*E. faecalis*	*E. faecalis*	*E. faecalis*	*E. faecalis*
Minimum inhibitory concentration (mg/L)	Linezolid	**4**	**4**	**4**	**4**
	Florfenicol	**64**	**16**	**16**	**32**
	Erythromycin	**>64**	**>64**	**>64**	1
	Tetracycline	**64**	**32**	**64**	<0.25
	Ampicillin	0.5	0.5	0.5	0.5
	Ciprofloxacin	0.5	0.5	0.5	0.5
	Vancomycin	2	1	2	2
Antimicrobial resistance genes		*optrA, poxtA, fexB, erm(B), tet(L), dfrG, lsa(A)*	*optrA, fexA, erm(A), tet(L), ant(6)-Ia, ant(9)-Ia, aph(3′)-III, cat, dfrG,lsa(A)*	*optrA, fexA, erm(A), erm(B), tet(L), ant(6)-Ia, ant(9)-Ia, aph(3′)-III, cat, dfrG, lsa(A)*	*poxtA, fexB, aac(6′)-aph(2″), lsa(A)*
ST		1071	506	32	960
Accession No.		SAMD00915482	SAMD00915483	SAMD00915484	SAMD00915485

Resistance is indicated in underlined bold. Intermediate is indicated in bold.

ST: Sequence type.

## 4 Discussion

Half of the RMBDs in commercial pet foods sold in Japan exceeded 10^6^ CFU/g of SPCs, whereas all heat-treated foods and treats had below 10^6^ CFU/g of SPCs. This suggests that RMBDs in pet foods had higher bacterial contamination levels than heat-treated foods and treats. SPC is one of the hygienic indicator of bacterial contamination, and the maximum acceptable level of which in feeds is 10^6^ CFU/g [29]. Reports from other countries have also shown that approximately 25–75 % of RMBDs for pets exhibit SPC levels exceeding 10^6^ CFU/g, whereas non-RMBDs have lower SPC levels [1,30]. Among the RMBDs tested, BARFs, which contained not only raw meats but also other ingredients, showed the highest SPC level, suggesting that bacterial contamination occurs during the manufacturing process of RMBDs, especially BARFs [30]. These results suggested that RMBDs have a risk of bacterial contamination at unacceptable levels, and using them as pet food is not recommended [2,18,31]. Pet owners should be alerted of this risk. Therefore, feeding RMBDs to pets should be approached with caution.

In the present study, *E. coli* and *Enterococcus* spp. were isolated from 62.5 % and 89.5 % of RMBDs, respectively, similar to previous findings (60–87 % and 100 %, respectively) [10,11,13]. *E. coli* and *Enterococcus* spp. are commonly used as indicator bacteria of fecal contamination. Most RMBDs were contaminated with *E. coli* or *Enterococcus* spp., indicating potential fecal contamination and suggesting that intestinal pathogens may be present in RMBDs, posing risks of adverse health issues in pets and owners [32]. *Enterococcus* spp. have been used as microbiological indicators of manufacturing sanitation, particularly in frozen food production, owing to their high tolerance to freezing conditions [33]. As many commercial RMBDs for pets are frozen, *Enterococcus* spp. is possibility to be an appropriate hygienic indicator. Standards and regulations for specific microorganisms such as hygienic indicator bacteria and pathogens have not yet been established in many countries, including Japan [7,18,34]. In the EU, the proposed regulations for pet foods require less than 300 CFU/g of Enterobacteriaceae and the absence of *Salmonella* spp. [35]. To reduce the risks to public health from pet foods, researching the bacterial contamination status of pet foods and establishing standards for hygienic indicator bacteria and pathogens are necessary to provide safe products.

*E. coli* strains harboring cephalosporin (*bla*) resistance genes and one of these isolate harboring colistin (*mcr*) resistance gene were detected in RMBDs, particularly in chicken meat products. *Enterococcus* spp. carrying mobile linezolid resistance genes (*optrA* and *poxtA*) were detected in RMBDs, particularly in BARF. The isolation of antimicrobial-resistant bacteria from RMBDs, especially those resistant to veterinary and human clinically important antimicrobials, has been previously reported [7]. These antimicrobial resistance genes are transferable across bacterial genera, including pathogenic bacteria [14,36]. Cephalosporin-resistant *E. coli* have been reported in raw chicken meat-based pet foods, and it would be risks of the antimicrobial-resistant bacteria contamination [12]. Multidrug-resistant bacteria were detected more frequently in dogs fed RMBDs than in those other fed conventional foods [15]. Additionally, the transmission of antimicrobial-resistant bacteria between pets and humans is an ongoing concern [16,37]. As a risk of disseminating antimicrobial-resistant bacteria via pet food to pets and their owners exists, feeding RMBDs to pets could be hazardous. Based on these results, feeding RMBDs to pets is discouraged because of the potential contribution to the spread of antimicrobial-resistant bacteria.

In this study, *Enterococcus* spp. were isolated from 23.3 % of treats. Pathogens such as *Salmonella* spp. have previously been isolated from non-RMBD pet food products, and outbreaks in both humans and pets caused by *Salmonella* spp.-contaminated pet foods have also been reported [3,4,9,38]. Treats are typically used as training rewards, fed directly from human hands to pets [39]. To minimize the risk of pathogen transmission from pet foods to humans and pets, enhancing the owner's knowledge and behavior regarding safe pet food handling, including washing the hands, is important in addition to reducing pathogens in pet food products [40].

In the present study, the SPC levels were low in heat-treated foods, and no *E. coli* or enterococci were isolated. Bacterial contamination and pathogen isolation have been reported in non-RMBDs used as pet food [1,41]. As temperature and water activity are one of important factors for reducing the abundance of microorganisms, thermal inactivation and drying are performed [42]. To remove pathogens from pet foods, identifying pathogen contamination routes during pet food production and setting appropriate sterilization conditions are needed.

In this study, we aimed to investigate the bacterial contamination of commercial mass-marketed pet food products sold in Japan. Despite its strengths, this study had several limitations. First, pet foods were purchased by mail order or from supermarkets that were mass-marketed products. However, pet foods are also sold through other channels, such as veterinary hospitals and local or specialty shops, including direct-to-consumer companies, and the bacterial contamination levels of these products could not be investigated [2,43]. Moreover, the origins of the bacteria that contaminated pet foods and their transmission to pets and humans could not be identified. In the future, it will be necessary to investigate these areas to improve the quality of pet foods and the wellness of pets and humans and to investigate a wide range of pet foods.

## 5 Conclusion

We clarified the actual situation of bacterial contamination, including antimicrobial-resistant bacteria, in various types of commercial pet foods in Japan. In RMBDs, high levels of bacterial contamination existed. Cephalosporin-resistant *E. coli* and linezolid-intermediate *E. faecalis*, which are important antimicrobial-resistant bacteria in veterinary and human clinical settings, were detected. In light of the present results, the practice of feeding RMBDs to pets is discouraged, in consistency with recent CDC warnings [2]. To prevent the transmission of microorganisms, including antimicrobial-resistant bacteria and pathogens, improving the quality of pet foods and owner awareness feeding about pet foods are necessary.

The following are the supplementary data related to this article.

Table S1 Information about pet foods used in this study. Table S1

## Data statement

Whole-genome sequencing data were deposited in DDBJ (BioProject accession number: PRJDB20756, BioSample accession number: SAMD00915481).

## CRediT authorship contribution statement

**Akira Fukuda:** Writing – review & editing, Writing – original draft, Visualization, Validation, Supervision, Resources, Project administration, Methodology, Investigation, Formal analysis, Data curation, Conceptualization. **Kazu Yamaura:** Writing – review & editing, Visualization, Validation, Formal analysis, Data curation. **Kazuyoshi Tokumoto:** Writing – review & editing, Supervision, Resources. **Yasuhiko Suzuki:** Writing – review & editing, Resources, Funding acquisition. **Chie Nakajima:** Writing – review & editing, Resources. **Shoichiro Yukawa:** Writing – review & editing, Validation, Investigation. **Masaru Usui:** Writing – review & editing, Supervision, Resources, Project administration, Methodology, Data curation, Conceptualization.

## Funding

This work was supported in part by the Japan Agency for Medical Research and Development (AMED) under the grant numbers JP20wm0125008 and JP253fa627005 to YS.

## Declaration of competing interest

None declared.

## Data Availability

Data will be made available on request.
